# Supplementary material for: Content-rich biological network constructed by mining PubMed abstracts
Source: BMC Bioinformatics. 2004 Oct 8;5:147. doi: 10.1186/1471-2105-5-147 (PMC528731; doi:10.1186/1471-2105-5-147)
Supplement: Additional File 2 — The original results of the above study (non-essential files are deleted to keep the file size under the limit set by BMC bioinformatics). [file 1471-2105-5-147-S2.bz2 › chilibotAdditionalFile2/dip20/674ID10767348E11080/html/ACVRL1_ENG.html]

 


 **ACVRL1** and **ENG** 
  
Found 524 abstracts in PubMed, retrieved 20.  
 

 What does Google say? 
 PDF only 
| .edu only 

---

**Interactive relationship** (e.g. stimulation, inhibition, etc)

**Neutral relationship**- Thromboxane A 2 synthase TXAS binds to the endoplasmic reticulum membrane and catalyzes both an isomerization of prostaglandin H 2 PGH 2 to form thromboxane A 2 TXA 2 and a fragmentation of PGH 2 to form 12 hydroxy, 10 heptadecatrienoic acid HHT  [ **ENG** ]   [ **ACVRL1** ]  and malondialdehyde MDA.  Ref: 12859980 Arch Biochem Biophys, 2003
- We found that HHT  [ **ENG** ]   [ **ACVRL1** ]  was metabolized to an acid product HHT  [ **ENG** ]   [ **ACVRL1** ]  acid.  Ref: 12724858 J Exp Ther Oncol< MedlineTA>J Exp Ther Oncol, 2003
- HHT  [ **ENG** ]   [ **ACVRL1** ]  types 1 and 2 are caused by loss of function mutations in  **ENG**  and  **ACVRL1** .  Ref: 12812985 Hum Mol Genet, 2003
- Activin receptor like kinase 1  [ **ACVRL1** ]  ALK 1  [ **ACVRL1** ] , the gene mutated in HHT  [ **ENG** ]   [ **ACVRL1** ]  type 2 HHT2  [ **ACVRL1** ] , is a serine threonine kinase receptor type I of the TGF beta superfamily, specifically expressed on endothelial cells.  Ref: 12700602 Eur J Hum Genet, 2003
- The two known disease types, HHT1  [ **ENG** ]  and HHT2  [ **ACVRL1** ] , are caused by mutations in the endoglin  [ **ENG** ]   **ENG**  and ALK 1  [ **ACVRL1** ]  genes, respectively.  Ref: 12843319 J Med Genet< MedlineTA>J Med Genet, 2003
- Should asymptomatic patients with hereditary haemorrhagic telangiectasia HHT  [ **ENG** ]   [ **ACVRL1** ]  be screened for cerebral vascular malformations ? Data from 22 061 years of HHT  [ **ENG** ]   [ **ACVRL1** ]  patient life.  Ref: 12754343 J Neurol Neurosurg Psychiatry, 2003
- In mice, HHT  [ **ENG** ]   [ **ACVRL1** ]  acid was detected in the plasma within 5 min of the i.v. injection of HHT  [ **ENG** ]   [ **ACVRL1** ]  and declined rapidly thereafter.  Ref: 12724858 J Exp Ther Oncol< MedlineTA>J Exp Ther Oncol, 2003
- Hereditary hemorrhagic telangiectasia HHT  [ **ENG** ]   [ **ACVRL1** ]  is a vascular disorder in humans which has been mapped to two genes, endoglin  [ **ENG** ]  and activin receptor like kinase 1  [ **ACVRL1** ]  ALK 1  [ **ACVRL1** ]  both of which mediate signaling by transforming growth factor beta ligands in vascular endothelial cells.  Ref: 12667943 Cardiovasc Res, 2003
- Twenty four hours after HHT  [ **ENG** ]   [ **ACVRL1** ]  dosing in mice, approximately 29% of the dose was excreted in the urine as HHT  [ **ENG** ]   [ **ACVRL1** ]  and 20% as HHT  [ **ENG** ]   [ **ACVRL1** ]  acid.  Ref: 12724858 J Exp Ther Oncol< MedlineTA>J Exp Ther Oncol, 2003

**Non-interactive relationship** (e.g. studied together, co-existance, homology, etc.)

- The HHT  [ **ENG** ]   [ **ACVRL1** ]  concentration inhibiting 50% of the growth of human leukemic HL 60 cells was 20 ng ml, while for HHT  [ **ENG** ]   [ **ACVRL1** ]  acid it was 1 00 ng ml, indicating that the acid form was more than 700 times less cytotoxic than HHT  [ **ENG** ]   [ **ACVRL1** ] .  Ref: 12724858 J Exp Ther Oncol< MedlineTA>J Exp Ther Oncol, 2003
- 1.05 to 2.99 yielded a haemorrhage rate in individuals with cerebral AV malformations of 1.4 2.0% per annum, comparable to figures in the non HHT  [ **ENG** ]   [ **ACVRL1** ]  cerebral AV malformation population.  Ref: 12754343 J Neurol Neurosurg Psychiatry, 2003
- The diagnosis of HHT  [ **ENG** ]   [ **ACVRL1** ]  consists of recurrent epistaxis, mucocutaneous telangiectasis, visceral vascular lesion and familial occurrence.  Ref: 12636208 Zhonghua Yi Xue Za Zhi Taipei , 2002
- When mice were given an intravenous i.v. injection of HHT  [ **ENG** ]   [ **ACVRL1** ]  4 mg kg, the HHT  [ **ENG** ]   [ **ACVRL1** ]  acid metabolite was found in both plasma and urine.  Ref: 12724858 J Exp Ther Oncol< MedlineTA>J Exp Ther Oncol, 2002
- Methods We reviewed the angiographic findings of seven patients with HHT  [ **ENG** ]   [ **ACVRL1** ] .  Ref: 12813620 Cardiovasc Intervent Radiol< MedlineTA>Cardiovasc Intervent Radiol, 2002
- Hereditary hemorrhagic telangiectasia HHT  [ **ENG** ]   [ **ACVRL1** ] , also known as Osler Weber Rendu disease, is a rare disorder characterized by arteriovenous communications in visceral organs.  Ref: 12636208 Zhonghua Yi Xue Za Zhi Taipei , 2002
- CONCLUSIONS These data contradict accepted wisdom in many countries that asymptomatic HHT  [ **ENG** ]   [ **ACVRL1** ]  patients are at a low and acceptable risk of haemorrhage.  Ref: 12754343 J Neurol Neurosurg Psychiatry, 2003
- These findings stress the need for an early diagnosis of HHT  [ **ENG** ]   [ **ACVRL1** ]  that can be useful for the early control of associated visceral involvement.  Ref: 12843319 J Med Genet< MedlineTA>J Med Genet, 2003
- The TXAS 2C5 chimera contains heme at nearly a 1 1 molar ratio and catalyzes the formation of TXA 2, MDA, and HHT  [ **ENG** ]   [ **ACVRL1** ]  at a 1 1 1 ratio, although with a reduced catalytic activity compared to wild type TXAS.  Ref: 12859980 Arch Biochem Biophys, 2003
- 12 HHT  [ **ENG** ]   [ **ACVRL1** ] , 13 78% inhibition and weaker inhibition on the formation of 12 HETE 0 49% inhibition.  Ref: 12781627 Toxicol Appl Pharmacol< MedlineTA>Toxicol Appl Pharmacol, 2003
- The results indicated that compared with the control, the level of hTERT mRNA synthesis in HL 60 cells treated with HHT  [ **ENG** ]   [ **ACVRL1** ]  at 0.005 0.03 micro g ml for 12 hours did not change significantly, but it reduced obviously at 0.04 micro g ml and was undetectable at 0.05 micro g ml.  Ref: 12744738 Zhongguo Shi Yan Xue Ye Xue Za Zhi, 2003
- Haemorrhages were also six times more common in female HHT  [ **ENG** ]   [ **ACVRL1** ]  subjects 6.18.  Ref: 12754343 J Neurol Neurosurg Psychiatry, 2003
- The identification of ALK 1  [ **ACVRL1** ]  mutations in subjects with a suspected diagnosis and without clinical signs of HHT  [ **ENG** ]   [ **ACVRL1** ]  argue in favour of a molecular diagnosis.  Ref: 12843319 J Med Genet< MedlineTA>J Med Genet, 2003
- The aim of this report is to highlight the biliary manifestations of HHT  [ **ENG** ]   [ **ACVRL1** ]  and draw attention to an unusual complication of ERCP in this setting.  Ref: 12783354 Endoscopy, 2003
- METHODS Retrospective study on stroke incidence in individuals with HHT  [ **ENG** ]   [ **ACVRL1** ]  and their immediate families n = 674.  Ref: 12754343 J Neurol Neurosurg Psychiatry, 2003
- Purpose To describe the angiographic features of hepatic involvement in hereditary hemorrhagic telangiectasia HHT  [ **ENG** ]   [ **ACVRL1** ] , particularly the presence of portovenous shunts.  Ref: 12813620 Cardiovasc Intervent Radiol< MedlineTA>Cardiovasc Intervent Radiol, 2003
- Vascular lesions of the gastrointestinal GI tract include arterio venous malformations as angiodysplasia and Dieulafoy s lesion, venous ectasias multiple phlebectasias and haemorroids, teleangiectasias which can be associated with hereditary hemorrhagic teleangiectasia HHT  [ **ENG** ]   [ **ACVRL1** ] , Turner s syndrome and systemic sclerosis, haemangioma s, angiosarcoma s and disorders of connective tissue affecting blood vessels as pseudoxanthoma elasticum and Ehlers Danlos s disease.  Ref: 12619428 Acta Gastroenterol Belg< MedlineTA>Acta Gastroenterol Belg, 2003
- This controlled, prospective study defined sonographic criteria for hepatic involvement in HHT  [ **ENG** ]   [ **ACVRL1** ] .  Ref: 12717395 Hepatology, 2003
- The diameter of the common hepatic artery was significantly dilated without overlap between HHT  [ **ENG** ]   [ **ACVRL1** ]  patients with liver involvement and the 3 control groups mean 11.3 2.8 mm HHT  [ **ENG** ]   [ **ACVRL1** ]  with liver involvement, 4.6 0.9 mm HHT  [ **ENG** ]   [ **ACVRL1** ]  without liver involvement, 4.8 1.0 mm cirrhosis, and 4.4 1.0 mm healthy controls, P < ;.  Ref: 12717395 Hepatology, 2003
- In conclusion, the transcription of hTERT mRNA in HL 60 cells can be inhibited by HHT  [ **ENG** ]   [ **ACVRL1** ] .  Ref: 12744738 Zhongguo Shi Yan Xue Ye Xue Za Zhi, 2003
- When HL 60 cells incubated with HHT  [ **ENG** ]   [ **ACVRL1** ]  at 0.02 micro g ml for various hours, the expression of hTERT mRNA did not decrease after 6 18 hours and reduced significantly after 24 hours and was undetectable at 30 hours.  Ref: 12744738 Zhongguo Shi Yan Xue Ye Xue Za Zhi, 2003
- Hepatobiliary manifestations of hereditary hemorrhagic telangiectasia HHT  [ **ENG** ]   [ **ACVRL1** ]  are rare, but often involve cholestasis.  Ref: 12783354 Endoscopy, 2003
- A variety of therapies have been proposed for epistaxis control in HHT  [ **ENG** ]   [ **ACVRL1** ]  but with limited success.  Ref: 12658738 Head Neck, 2003
- Hepatic involvement in hereditary hemorrhagic telangiectasia HHT  [ **ENG** ]   [ **ACVRL1** ]  is highly variable and may lead to severe clinical symptoms such as heart failure.  Ref: 12717395 Hepatology, 2003
- The Harmonic Scalpel may be the treatment of choice for recurrent epistaxis in HHT  [ **ENG** ]   [ **ACVRL1** ] .  Ref: 12658738 Head Neck, 2003
- BACKGROUND The frequency of haemorrhage in individuals with hereditary haemorrhagic telangiectasia HHT  [ **ENG** ]   [ **ACVRL1** ] , 10% of whom will have cerebral arteriovenous AV malformations, could be high enough to justify screening.  Ref: 12754343 J Neurol Neurosurg Psychiatry, 2003
- 22 061 HHT  [ **ENG** ]   [ **ACVRL1** ]  patient years, specifically analysing patients under 46 years of age 17 515 patient years.  Ref: 12754343 J Neurol Neurosurg Psychiatry, 2003
- Color Doppler sonography and pulsed Doppler sonography were used to study 25 patients with HHT  [ **ENG** ]   [ **ACVRL1** ]  and liver involvement, 20 patients with HHT  [ **ENG** ]   [ **ACVRL1** ]  without liver involvement, 25 patients with cirrhosis, and 25 patients without liver disease.  Ref: 12717395 Hepatology, 2003
- Conclusions Hepatic vascular lesions in HHT  [ **ENG** ]   [ **ACVRL1** ]  are varied, ranging from telangiectasias to large shunts between three vascular channels.  Ref: 12616420 Cardiovasc Intervent Radiol< MedlineTA>Cardiovasc Intervent Radiol, 2003
- We report two cases of HHT  [ **ENG** ]   [ **ACVRL1** ]  in which recurrent nasal bleeding was successfully controlled using the Harmonic Scalpel.  Ref: 12658738 Head Neck, 2003
- Autosomal dominant hereditary haemorrhagic telangiectasia HHT  [ **ENG** ]   [ **ACVRL1** ]  is a genetically heterogeneous disease caused by mutations in at least two different loci.  Ref: 12786761 Clin Genet< MedlineTA>Clin Genet, 2003
- Hereditary haemorrhagic telangiectasia HHT  [ **ENG** ]   [ **ACVRL1** ]  is a genetic vascular disorder characterised by epistaxis, telangiectases, and visceral manifestations.  Ref: 12843319 J Med Genet< MedlineTA>J Med Genet, 2003
- Homoharringtonine HHT  [ **ENG** ]   [ **ACVRL1** ] , first isolated from the Chinese evergreen Cephalotaxus harringtonia, has been demonstrated to have a broad antitumor activity in rodents and antileukemic effects in humans.  Ref: 12724858 J Exp Ther Oncol< MedlineTA>J Exp Ther Oncol, 2003
- Substrate specificity of the alpha L arabinofuranosidase from Rhizomucor pusillus HHT  [ **ENG** ]   [ **ACVRL1** ]  1.  Ref: 12829392 Carbohydr Res, 2003
- A higher frequency of pulmonary arteriovenous malformations AVMs has been reported for HHT1  [ **ENG** ]  while HHT2  [ **ACVRL1** ]  is thought to be associated with a lower penetrance and milder disease manifestations.  Ref: 12843319 J Med Genet< MedlineTA>J Med Genet, 2003
- In hereditary hemorrhagic telangiectasia HHT  [ **ENG** ]   [ **ACVRL1** ] , the hallmark vascular lesion is termed an arteriovenous malformation, which involves the direct communication of an artery with a vein arteriovenous shunt, without an intervening capillary bed.  Ref: 12668602 Hum Mol Genet, 2003
- The relationship between regulation of hTERT mRNA expression and HHT  [ **ENG** ]   [ **ACVRL1** ]  induced apoptosis is worth investigating furtherly.  Ref: 12744738 Zhongguo Shi Yan Xue Ye Xue Za Zhi, 2003
- The prevalence of liver involvement of HHT  [ **ENG** ]   [ **ACVRL1** ]  was reported to range from 8 to 31%.  Ref: 12636208 Zhonghua Yi Xue Za Zhi Taipei , 2002
- In all patients with HHT  [ **ENG** ]   [ **ACVRL1** ]  and liver involvement, areas with intrahepatic hypervascularization caused by dilated intrahepatic arteries were observed in varying intensity.  Ref: 12717395 Hepatology, 2003
- Purpose To describe the angiographic features of hepatic involvement in hereditary hemorrhagic telangiectasia HHT  [ **ENG** ]   [ **ACVRL1** ] , particularly the presence of portovenous shunts.  Ref: 12616420 Cardiovasc Intervent Radiol< MedlineTA>Cardiovasc Intervent Radiol, 2003
- The tendency for suppression of telomerase activity was consistent with decrease of the expression of hTERT mRNA in HHT  [ **ENG** ]   [ **ACVRL1** ]  treated HL 60 cells.  Ref: 12744738 Zhongguo Shi Yan Xue Ye Xue Za Zhi, 2003
- In conclusion, the diameter of the common hepatic artery >7 mm and intrahepatic hypervascularization are suitable sonographic diagnostic parameters of HHT  [ **ENG** ]   [ **ACVRL1** ]  with high sensitivity and specificity.  Ref: 12717395 Hepatology, 2003
- The initial half lives t 1 2 alpha of HHT  [ **ENG** ]   [ **ACVRL1** ]  and HHT  [ **ENG** ]   [ **ACVRL1** ]  acid were 9 and 17 min, respectively.  Ref: 12724858 J Exp Ther Oncol< MedlineTA>J Exp Ther Oncol, 2003
- The lethal dose of HHT  [ **ENG** ]   [ **ACVRL1** ]  affecting 50% LD50 of mice was 6.7 mg kg, but HHT  [ **ENG** ]   [ **ACVRL1** ]  acid produced no apparent toxic effects at doses up to 280 mg kg.  Ref: 12724858 J Exp Ther Oncol< MedlineTA>J Exp Ther Oncol, 2003
- Herein, we present a 75 year old male who was diagnosed as having HHT  [ **ENG** ]   [ **ACVRL1** ]  with liver involvement, based on the findings of recurrent epistaxis, mucosal telangiectasis on the lower lip and hepatic arteriovenous malformation.  Ref: 12636208 Zhonghua Yi Xue Za Zhi Taipei , 2002
- The transforming growth factor beta signaling family is a key player in genetic and multifactorial diseases, including hereditary hemorrhagic telangiectasia HHT  [ **ENG** ]   [ **ACVRL1** ] , cancer, atherosclerosis and immunomodulation.  Ref: 12812985 Hum Mol Genet, 2003
- Methods We reviewed the angiographic findings of seven patients with HHT  [ **ENG** ]   [ **ACVRL1** ] .  Ref: 12616420 Cardiovasc Intervent Radiol< MedlineTA>Cardiovasc Intervent Radiol, 2003
- Boiled plasma prevented the conversion of HHT  [ **ENG** ]   [ **ACVRL1** ]  to HHT  [ **ENG** ]   [ **ACVRL1** ]  acid, suggesting that the conversion was enzymatically mediated.  Ref: 12724858 J Exp Ther Oncol< MedlineTA>J Exp Ther Oncol, 2003
- We report here a case of HHT  [ **ENG** ]   [ **ACVRL1** ]  associated with cholestasis due to common bile duct stenosis.  Ref: 12783354 Endoscopy, 2003
- To explore the possible actions of endocrine disruptors on the autacoid synthesis in the body, we investigated the effects of nonylphenol NP, bisphenol A BPA, di n butyl phthalate DBP, benzyl n butyl phthalate BBP, and di 2 ethylhexyl phthalate DEHP on the formation of 12 lipoxygenase metabolite, 12 HETE, and cyclooxygenase metabolites, TXB 2 and 12 HHT  [ **ENG** ]   [ **ACVRL1** ] , from exogenous arachidonic acid AA in rabbit platelets.  Ref: 12781627 Toxicol Appl Pharmacol< MedlineTA>Toxicol Appl Pharmacol, 2003
- To investigate the mechanism and its significance of the depression of telomerase activity in HL 60 cells exposed to homoharringtonine HHT  [ **ENG** ]   [ **ACVRL1** ] , the semi quntitative RT PCR and PCR ELISA were used to detect the expression of hTERT mRNA and telomerase activity in HL 60 cells, the apoptotic rate of HL 60 cells was assayed with TUNEL.  Ref: 12744738 Zhongguo Shi Yan Xue Ye Xue Za Zhi, 2003
- For the first time the parental germline in which a de novo HHT  [ **ENG** ]   [ **ACVRL1** ]  mutation occurred has been identified.  Ref: 12786761 Clin Genet< MedlineTA>Clin Genet, 2003
- A preliminary investigation was performed to exclude association between the predisposition and certain candidate genes including KDR kinase insert domain receptor, TEK TEK tyrosine kinase endothelial,  **ACVRL1**  activin receptor like kinase 1  [ **ACVRL1** ] ,  **ENG**  endoglin  [ **ENG** ]  and FLT4 fms like tyrosine kinase 4.  Ref: 12752572 Clin Genet, 2003
- Sonographic criteria for the diagnosis of hepatic involvement in hereditary hemorrhagic telangiectasia HHT  [ **ENG** ]   [ **ACVRL1** ] .  Ref: 12717395 Hepatology, 2003
- High pressure liquid chromatography and mass spectrometry were used to confirm the identity and quantify HHT  [ **ENG** ]   [ **ACVRL1** ]  and its metabolite, HHT  [ **ENG** ]   [ **ACVRL1** ]  acid.  Ref: 12724858 J Exp Ther Oncol< MedlineTA>J Exp Ther Oncol, 2003
- The alpha L arabinofuranosidase AF from the fungus Rhizomucor pusillus HHT  [ **ENG** ]   [ **ACVRL1** ]  1 released arabinose at appreciable rates from 1 >5 alpha L arabinofuranooligosaccharides, sugar beet arabinan and debranched arabinan.  Ref: 12829392 Carbohydr Res, 2003
- Conclusions Hepatic vascular lesions in HHT  [ **ENG** ]   [ **ACVRL1** ]  are varied, ranging from telangiectasias to large shunts between three vascular channels.  Ref: 12813620 Cardiovasc Intervent Radiol< MedlineTA>Cardiovasc Intervent Radiol, 2003
- Overcorrecting for any bias towards overestimation that would be introduced in excluding non penetrant family members, cerebral haemorrhages were more than 20 times more common in male HHT  [ **ENG** ]   [ **ACVRL1** ]  subjects under the age of 45 years than in the general population standardised ratio 22.99.  Ref: 12754343 J Neurol Neurosurg Psychiatry, 2003
- HHT  [ **ENG** ]   [ **ACVRL1** ]  can be definitely diagnosed with the presence of all these three criteria.  Ref: 12636208 Zhonghua Yi Xue Za Zhi Taipei , 2002
- OBJECTIVES To define the annual incidence of haemorrhagic stroke in individuals with HHT  [ **ENG** ]   [ **ACVRL1** ] .  Ref: 12754343 J Neurol Neurosurg Psychiatry, 2003
